# Patterns and Determinants of Treatment Seeking among Previously Untreated Psychotic Patients in Aceh Province, Indonesia: A Qualitative Study

**DOI:** 10.1155/2016/9136079

**Published:** 2016-06-12

**Authors:** Marthoenis Marthoenis, Marion C. Aichberger, Meryam Schouler-Ocak

**Affiliations:** ^1^University Psychiatric Clinic of Charité at St. Hedwig's Hospital, Große Hamburger Strasse 5-11, 10115 Berlin, Germany; ^2^Departments of Psychiatry and Psychotherapy, Charite Universitätsmedizin, Campus Charité Mitte, Charitéplatz 1, 10117 Berlin, Germany

## Abstract

Immediate treatment of first-episode psychosis is essential in order to achieve a positive outcome. However, Indonesian psychiatric patients often delay accessing health services, the reason for which is not yet fully understood. The current study aimed to understand patterns of treatment seeking and to reveal determinants of the delay in accessing psychiatric care among first-time user psychotic patients. Qualitative interviews were conducted with sixteen family members who accompanied the patients to a psychiatric hospital. Many families expressed beliefs that mental illness appertains to* village sickness* and not* hospital sickness*; therefore, they usually take the patients to traditional or religious healers before taking them to a health professional. They also identified various factors that potentially delay accessing psychiatric treatment: low literacy and beliefs about the cause of the illness, stigmatisation, the role of extended family, financial problems, and long distance to the psychiatric hospital. On the other hand, the family mentioned various factors related to timely help seeking, including being a well-educated family, living closer to health facilities, previous experience of successful psychotic therapy, and having more positive symptoms of psychosis. The findings call for mental health awareness campaigns in the community.

## 1. Introduction

Seeking treatment is a continuous process whereby individual symptoms of illness develop and are gradually noticed and are evaluated and finally treated through the initiation of specific interventions [1–3]. The outcome of this treatment is later used by the patient and the family in order to develop their own explanation, this becoming the basis of further treatment seeking decisions [2]. The process of seeking treatment is repeated until the goal of recovery is met. The early symptoms of mental disorders are not always noticeable to lay people; the seeking of treatment for a psychosis is therefore sometimes delayed.

Immediate treatment of first-episode psychosis is critical in order to achieve a positive treatment outcome [4, 5]. First-episode psychosis is the time when an individual begins to experience psychosis for the first time [6]. Psychiatric treatment must be obtained as soon as possible after the onset of the first episode. Delays to the onset of treatment prolong the duration of untreated psychosis (DUP). A longer DUP is associated with poorer treatment outcomes [7], while a shorter DUP is associated with better treatment outcomes [8].

Several factors have been found to be related to the initiation of psychiatric treatment. Early age at onset, type of diagnosis, and poor premorbid adjustment during adolescence were associated with a longer DUP [9]. Familial and societal beliefs about mental illness and fear of labelling are also related to delayed treatment [10]. Among Southeast Asian populations, for example, in Cambodia, low literacy on mental disorders and inappropriate lay beliefs about the cause hindered timely help seeking [11]. Similarly, attitudes and beliefs about supernatural causes of mental illness were also crucial factors in the pathway to care [12]. On the other hand, several factors may lead to early treatment seeking. Examples include family strength [13], family involvement in seeking mental health care [14], previous family experience with mental illness [15], and primary positive symptoms of psychotic or aggressive-violence behaviour [10], all of which have been connected to early help seeking. Whether factors delay or accelerate the treatment initiation, these factors function at different levels. Aggressive behaviour can be categorised as being on the personal level, while a society's beliefs about mental illness can be regarded as being on the community level. Qualitative information from these different levels is expected to help improve the initiation of mental illness treatments in the community.

Moreover, despite the large psychiatric treatment gaps [16], longer DUP [17], and high burden of mental disorders in Indonesia [18], patterns and determinants of delayed treatment seeking among psychiatric patients in this country have rarely been studied. This study, therefore, aimed to qualitatively examine patterns of treatment seeking and to understand the determinants of its delay among those with a psychosis who had never been professionally treated in the province of Aceh, Indonesia.

## 2. Methods

This study applied a qualitative approach. A qualitative approach allows the researcher to discuss in detail the various contours of the issue [19], in this case determinants of delayed treatment seeking. The processes from the point at which the family recognises that the patient has started to think, talk, and behave differently from normal until the point at which they begin to seek mental health treatment can also be researched using this approach. The study was approved by the Medical Research Ethics Committee of the Medical Faculty at Syiah Kuala University in Banda Aceh. A research permit was also obtained from the Director of the Aceh Psychiatric Hospital.

### 2.1. Respondent Recruitment

Sixteen family members of seventeen patients who accompanied the patients to the hospital were invited to participate in this study. The inclusion criteria for the respondents were being 18 years or above, speaking either Bahasa Aceh or Bahasa Indonesia, and hospital admission because of a psychotic problem. Most patients were brought to the hospital by two to four family members, but only one family member of each patient was invited to participate in the study. Furthermore, one respondent (R16) came with two patients (P16 and P17). Informed consent was obtained from all of the respondents.

### 2.2. Data Collection

Data were collected via in-depth interviews, carried out by the first author (Marthoenis Marthoenis). The interviews were conducted from March to May 2014 at the outpatient clinic of the Aceh Psychiatric Hospital in Banda Aceh. The hospital is the only mental hospital in Aceh Province and is also the referral centre for psychiatric problems serving a population of around five million in the province.

A set of questions to collect sociodemographic information about the patient and the respondent were employed before the in-depth interview. Nine interviews were audio-taped for further analysis, while the remaining seven respondents did not agree to audio-taping and were thus recorded by note-taking only. Hesitation to be audio-recorded is common among the Acehnese; the experience of forced interrogation during military conflict was suggested as a possible reason for this reluctance.

Each respondent was asked to estimate the duration of untreated psychosis (DUP), that is, the time from when the patient started to behave differently, showing either positive or negative symptoms of psychosis, until the moment when the family took the patient to the hospital. The interviews started by asking the respondents to describe the patient's condition that had led to hospital admission. The interviews then focused on exploring the following questions based on the respondents' perspectives: (1) Which illness does the patient suffer from? (2) What causes the illness? (3) Before the family took the patient to the psychiatric hospital, where had they taken the patient for treatment? And finally (4) why did the family eventually bring the patient to the psychiatric hospital? The interviews were conducted either in Bahasa Aceh (Acehnese language) or in Bahasa Indonesia (Indonesian language), or a mix of both, according to the respondents' preference. Both of the languages are the native languages of the interviewer. Data from both audio-recording and note-taking were used for further analysis.

### 2.3. Analysis

A qualitative content analysis procedure suggested by Graneheim and Lundman [20] was used to analyse the data. A four-step analysis process was applied, starting by reading the notes and transcriptions several times in order to obtain the impression of the data. Afterwards, aim-related content units were identified and brought together into one unit of analysis. Content units that occurred frequently were then labelled with a code. The codes were then classified into subthemes based on their similarities and differences. Lastly, latent contents of the text were formulated as the themes [20].

## 3. Results

### 3.1. Demographic Information

Of the sixteen respondents interviewed, more than half (*n* = 9) were the parents of patients, five were siblings or cousins, one was the son, and one was the spouse. Most (*n* = 14) of the respondents were married, and half of the respondents (*n* = 8) were women. Six respondents had attended primary school only; four had completed senior high school, four had attended the university, one had graduated from junior high school, and another did not attend any formal education. Five respondents worked as farmers or fishermen, four as salesmen/saleswomen, three as housewives, two as government employees, and the other two as pensioners. Thirteen respondents were Acehnese, and three were Gayonese. All respondents were Muslim. The respondents' ages ranged from 27 to 68 years (average: 47 years old).

Of the seventeen patients who had come to the psychiatric hospital for the first time, sixteen had been diagnosed with schizophrenia and one with psychotic depression. The majority (*n* = 14) of patients were male. Ten of the patients were unmarried, three were married, and four were widowed. Two patients had never attended any formal education, five had completed primary school, four each had graduated from secondary school, and two had attended university. The majority (*n* = 12) of patients live in rural areas. Fourteen patients had seen a traditional or religious healer at least once for their mental problems, while only eight had seen a neurologist. Nine male patients were active smokers, three of whom had a history of cannabis use. The distance from the patients' village to the psychiatric hospital ranged from one to sixteen hours, travelling by public transport (average: 5.4 hours). The sample DUP ranged from two months to sixteen years. The patients' ages ranged from 14 to 74 years (average: 30.4 years).

### 3.2. Patterns of Treatment Seeking

The majority of patients in the current study shared a relatively similar pattern of treatment seeking behaviour. Whenever someone started to behave differently, showing either positive or negative symptoms of psychosis, the family often perceived it as a strange behaviour.

*His weird behaviour started about three years ago. Initially, we thought that was just his behaviour, because he was still able to attend university, so we just let him be like that. (*R*10)*

Whenever the patient's behaviour deteriorated, however, the family began to seek information about the problem by asking the neighbours, villagers, religious leaders, or community leaders. Traditional treatment usually started at this point. The family sometimes took the patient to traditional healers in their area. People who live in a rural farming area usually went to the traditional healer called* dukun* or to a religious healer locally known as* teungku*.

*The villagers said that he (her son – the patient) suffered from *teumamong*  (*possessed*), they insisted I should see a *dukun*[…] we have seen several *dukun* and *teungku* already. (*R*5)*

On the other hand, those living in a rural fishing area tended to take the patient to a* pawang laot*, a traditional fishermen community leader.

*…Of course, we saw [took him to] *pawang* *laot*, and he got better, but then he relapsed again and again, then we took him to another *pawang*, (and he was) good for some time, and then relapsed again, it was always like that…. (*R*12)*

If the treatments by any of those healers improved the patient's condition, the treatment was usually ceased. Nevertheless, many of the mental problems continued among the patients and their families continued to search for effective treatment.

*My brother has taken him (the patient) to (different) *dukun* twice, I have taken (him) three times, the parent has also taken him somewhere else several times. We gave up (with *dukun*) already, now we try here (to the psychiatric hospital). (*R*1)*

Only when the family felt that all of those treatments had progressed less successfully than hoped did they approach a health professional. The doctor or nurse at the health post who diagnosed the patient as suffering from a mental disorder usually referred the patient directly to the psychiatric hospital.

*…When we took her to the *puskesmas* (health post). The nurse asked me directly to take her here (to the psychiatric hospital). (*R*16)*

### 3.3. Determinants of Treatment Seeking

Treatment at psychiatric hospital seems to be the last option when treatment through the traditional sectors has not improved the patient's condition. This lengthy process of treatment seeking was not only responsible for the delay in psychiatric treatment but also time-consuming and caused the family to suffer financially. The long process of seeking psychiatric treatments might be caused by several factors such as the misattribution of the cause of mental illness, perceived stigma, financial problems, the long distance to the psychiatric hospital, and the complicated bureaucratic system (see Table 1).

#### 3.3.1. Theme 1: Misattribution of the Cause and Symptom of Mental Disorders

Delays in accessing mental health services were heavily driven by the low literacy of the family members regarding mental disorders. The majority of them believed that mental illness was caused by a supernatural power such as a ghost, black magic, being possessed, sorcery, or simply having a spell put on one by others. The supernatural causes of an illness were considered as* sakit kampung* (sakit = sick, kampung = village) by most of the family members.

*He had *sakit* *kampung*, he saw a pig or a dog on someone, then he hit him, otherwise he would not do that…. (*R*2)*

Belief about mental disorder being* sakit kampung* leads them to* berobat kampung* (berobat = to seek medication), seeking treatment by traditional healers.

*…Because we thought that (mental illness) was a *sakit* *kampung* and we did not know that was a *sakit* *rumahsakit* (hospital sickness), we thought *dukun* or *teungku* was a right person to treat the *sakit* *kampung*, right? (*R*10)*

The family members' beliefs about the cause of mental illness also varied depending on their residential area. Those who lived in a rural farming area explained that the illness was simply caused by black magic or spells, while a family member living in a rural fishing area related his son's illness to the supernatural power of the sea.

*I live in a fishing village, I do not know why it is, if the villagers said that he has *hantu* *laut* (*hantu* = ghost, *laut* = sea), then that is what I believe. (*R*12)*

These beliefs also determine the different traditional healers from whom help is sought, as was explained previously in the Patterns of Treatment Seeking.

Furthermore, since most of the family members use traditional healers as the first source of information, they had often been misinformed: sometimes, the healers not only provided inappropriate information about mental disorders, but also prevented the families from taking the patient to a health professional.

*…We went to see a *dukun* and he said that he was spelled black magic by someone, he did not allow us to see a doctor, because he said doctor gives pills that block the nerves…. (*R*5)*

Inappropriate information about patients' behaviour was also obtained from others. A mother, for instance, was convinced by local villagers that her son was suffering from malaria.

*When someone is smiling or talking to himself like he (the patient) is, the villagers said that is malaria. (*R*4)*

Besides the tendency to seek treatment from traditional healers, many family members brought patients to the neurologist, as they believed that mental disorder was part of a “sakit saraf” (*saraf* = nerve), and thus sought treatment by a* dokter saraf* (nerve doctor), the Indonesian word for neurologist.

*Usually people say *sakit* *saraf*, so we went to *dokter* *saraf*…. (*R*11)*

Another important issue that determines the promptness of seeking treatment was that of which symptoms of mental disorder a patient had. If a psychotic patient had predominantly positive symptoms, the family would acknowledge it straightforwardly and seek treatment much sooner.

*…When he returned home, he started to *mengamuk* (go amok) and was getting worse, unpredictable, might hit anyone….  (*R*1)*

*…He hit his mother few days ago, then I thought he has that (mental) problem, that is why we took him here…. (*R*10)*

On the other hand, patients with predominantly negative symptoms might be less likely to be considered as having a mental illness, and thus the impulse to seek treatment might be delayed.

*I was not sure if this is a mental illness, I thought it was a normal behaviour, I thought he was just lazy, that was why I did not take him here (to the psychiatric hospital), but I didn't know why he is so lazy and so slow. (*R*13)*

#### 3.3.2. Theme 2: Perceived Stigma

The perceived stigma of having a family member with a mental disorder is very prevalent in the Indonesian society, but the level of stigma differed between the families. Some families felt highly stigmatised and tried to hide the patients' illness from the neighbours.

*Are we not shy? We hid from our neighbours when we took him here. Look at what he is wearing right now… which father wouldn't be shy of seeing his son like this? (*R*10)*

Other family members who consider the illness to be God-given and have a positive attitude towards the treatment usually perceive lower stigma.

*Am I shy? No, not at all, because the illness is from Allah, we do not want to suffer with an illness, but if He gives it to us, what should we do? […] *Ikhtiyeu* is an obligation for muslims! (*R*14)*

*Ikhtiyeu* in Bahasa Aceh or* Ikhtiar* in Bahasa Indonesia literally means* initiative* but in this context would mean the efforts from the patients or the family to seek treatment.

Perceived stigma around mental illness might have profound consequences for the family's integrity. Many patients were left by their partner due to their mental problems.

*She was once married, but got divorced, I think because of her mental problem, the husband left her. (*R*3)*

#### 3.3.3. Theme 3: Role of Extended Family

Family members play a significant role in a patient's treatments. With regard to the patient's symptoms, the family seeks information on proper treatment, makes decisions about which hospital or health professional to take the patient to, and, most importantly, provides financial support. As an information seeker, the family usually asks someone who they think will know better about the patient's condition. After a family member has been convinced to try professional treatment, it sometimes takes time until the whole extended family agrees to take the patient to the psychiatric hospital.

*…You know, I asked everywhere, but I could not really come up with the answer. Then I met the ambulance driver, he said he often takes mental patients here [to the psychiatric hospital], he explained what to do, then I spoke to my family and we took him [the patient] here. (*R*15)*

The fact that the treatment seeking decision sometimes requires agreements from the extended family was also responsible for the delay in starting the psychiatric treatment. The eldest person in the family usually makes the final decision. In the case of P11, for instance, even though the patient had suffered from the illness for a considerably long time, the extended family could only take her to the hospital when her mother permitted them to do so.

*We wanted to take her to the doctor a long time ago, but her mother never allowed us. Now the mother is fed up as her behaviour is getting worse, so now she allowed us to…Only after the mother asked and her brother [agreed], we took her here. (*R*11)*

Additionally, the majority of psychiatric patients were unemployed. They therefore depend financially on their family.

#### 3.3.4. Theme 4: Financial Issues

A large number of families consider financial shortages as the cause of delayed hospital treatment. Some of them ask for money from other family members, while others run into debt to pay for the patient's treatment.

*Her husband did not want to go to the hospital, he said if I go there with you, that will need more money. You know, sir, we have wanted to take them here for a long time, but [they] have no money, how could we get here? (*R*16)*

Many families were aware that all consultation and treatments at the Aceh Psychiatric Hospital are free of charge as they are covered by the national health insurance for the poor. Nevertheless, they usually complained about transportation costs from their village to the hospital, for which many of them could not afford to pay.

*…It took 180 [rupiah] one-way with L300 [a type of public transport in Aceh] from there [their village] to here [Banda Aceh], the return is 360, that was for one person. Now three persons, at least we need…how much? One million [rupiah]? And [the cost for] our stay here? Where can we get that money, son? We know it [the treatment cost] is free here, but for our transport? (*R*2)*

At the same time, families with positive attitudes and strong hopes about the healing of the patients consider financial issues as the second challenge. They are more concerned about a good treatment outcome than about treatment costs.

*Money is an issue, but we will take him anywhere - everywhere, as long as he can get well. (*R*12)*

#### 3.3.5. Theme 5: Distance to the Psychiatric Hospital

Another important theme that had a negative impact on the speed of treatment seeking was the long distance from the patient's home to the psychiatric hospital. Respondents from the central part of Aceh more often expressed concern about the long duration of the journey than respondents living in districts closer to the psychiatric hospital.

*We departed from there yesterday after *ashar* praying, arrived here after the sunrise, so how long is that? 15 hours? 16? You count! […] That was a long journey right? And if we have to come here every time to get medicine, we would have no more time to work in the garden. (*R*3)*

A respondent who lives on a remote island also mentioned the lack of transportation available from his home to Banda Aceh. The community living in this area only has the option of renting a fishing boat to go to Banda Aceh.

*There are about one or two fishing boats that come here [to Banda Aceh from their village in the Pulo Aceh Island], if I miss that one, I will have to stay here [in Banda Aceh] again. (*R*8)*

Nevertheless, the distance to the psychiatric hospital did not contribute significantly towards delays in help seeking. In the case of P11, for instance, despite the fact that she lived in Banda Aceh area and the hospital was less than one hour away, she had suffered mental disorders for 16 years until her first contact with a psychiatrist.

#### 3.3.6. Theme 6: Perceived Complicated Bureaucratic System

A number of families criticised the complicated referral system prior to hospital admission. Usually, they had to obtain a reference letter from the district health post to which the patient is affiliated. Only with that letter and other documents can the patient be admitted to hospital.

*Early this morning, I took the *labi* − *labi* [a type of public transport] to here from the village, but when I arrived here, they said that I did not have the *Jamkesmas* [insurance] card, then I had to go home again to pick up the card, and then I came back here, thank God that they were still open and we could see the doctor […] This is too complicated for me, four hours on the road alone, we cannot understand this thing [system]…. (*R*12)*

## 4. Discussion

Treatment of mental disorders in the Acehnese society does not generally start as soon as an individual experiences positive or negative symptoms of psychosis. People usually consider these symptoms as a part of the patient's normal behaviour initially. Low literacy within the community regarding the symptoms of mental illness is therefore proposed as one of the determinants of delayed seeking of treatment for mental illness in this population. There has also been concern among mental health researches and services about issue of delayed psychiatric treatment as a result of low literacy [21].

Beliefs about supernatural cause of mental illness lead family members to take the patients to traditional healers. This issue is also frequently found in other developing countries [11, 22, 23] or even in developed countries such as in Singapore [12]. Treatments by traditional healers might be repeated several times, and consultation with a health professional was only sought when the family felt that the treatments with the traditional healer had not improved the patient's condition. The role of traditional healers in this kind of community is therefore influential [24]. Education for the family should also be initiated, since they are the first ones to recognise the change of the individual's behaviour.

The involvement of the family member in help seeking is persistent among Asian cultures [25]. They decide on the treatment and provide financial support during the treatment [26]. The role of the family should therefore be taken into consideration when addressing issues connected to treatment initiation.

With regard to the clinical symptoms of psychosis, positive symptoms help to initiate treatment seeking, while negative symptoms delay it. This verdict was in line with previous research [11], where positive symptoms of psychosis initiate the immediate seeking of treatment and negative symptoms delay the seeking of treatment.

Furthermore, treatment by a neurologist appears to be more acceptable to the Acehnese than treatments by a psychiatrist; despite the fact that consultation with a neurologist in their private clinic is more expensive than consultation with a psychiatrist, it is less stigmatised and in fact mentioned by some respondents with pride.

## 5. Conclusion

With reference to the respondents of this study, factors that potentially contribute towards the delay in treatment of psychotic patients in Aceh include misguided beliefs about the cause of the mental disorder, low literacy on the symptoms of mental disorders, internalised stigma, financial problems, the long distance to the psychiatric hospital, and the perceived complexity of the bureaucratic system. On the other hand, several conditions that catalyse help seeking are familiarity with mental illness, a higher level of education, better access to health services, and previous experience with successful therapies.

The long road to treatment experienced by this population reflects their hopes and expectations of recovery from a mental disorder. The individuality of treatment in each patient's path towards seeking treatment is revealed by the wide range of DUP, from two months to 16 years in this sample. This journey could be reduced if the mental health services were more accessible, family members were better informed, and less stigma existed towards mental illness. Further mixed-method studies are needed in order to unveil the role of culture, literacy, infrastructures, and the political system in the treatment seeking behaviours of psychiatric patients in other parts of Indonesia. The studies should obtain information generated from wider sources, rather than only from those who had already contacted the professional health services, as was the case in the current study.

## Figures and Tables

**Table 1:** Selected codes and categories that emerged while exploring the patterns and determinants of treatment seeking.

Selected codes	Theme
Patterns of treatment seeking	
Visiting traditional or religious healers	Repeated within traditional or religious healers before visiting the professional
Revisiting traditional or religious healers	
Unsatisfaction with the treatments	
Obtaining information from friends or neighbours	
Visiting health professionals	

Determinants of treatment seeking	
False belief about the cause	Misattribution of the cause and symptom of mental disorders
Being misinformed about the illness	
Inappropriate source of information	
Low literacy about the symptoms	

Feeling ashamed	Perceived stigma
Hiding the illness from others	

The whole family deciding on treatment seeking	Role of extended family
Dominant final decision from parent	
Patients depending on their family financially	

Unemployed patient	Financial issue
Poverty of the patient's family	
Expensive transportation cost to the hospital	
Limited coverage of health insurance	

Complaints about long distance to the hospital	Distance to the hospital
Limited transportation option from remote areas	

Confusion with referral system	Complicated bureaucratic system
Complaints with admission system	

## Additional Points

Despite the fact that the information from the respondents fulfilled the intentions of this study, the sample size was relatively small and therefore not representative of the Acehnese or the Indonesian in general. Secondly, information was obtained only from those families who visited the psychiatric hospital, leaving us without information on those who are at home and have never been to the hospital.
